# The Effect of Partial and Temporary Vaccination on African Swine Fever Eradication Rates

**DOI:** 10.1155/2024/9409991

**Published:** 2024-01-17

**Authors:** Vincenzo Gervasi, Vittorio Guberti

**Affiliations:** Istituto Superiore per la Protezione e la Ricerca Ambientale, Ozzano Emilia, Bologna, Italy

## Abstract

African swine fever (ASF) is one of the most severe diseases of pigs, with drastic impact on pig industry. Wild boar populations play the role of ASF virus epidemiological reservoir. No effective and safe ASF vaccine is available, yet, but a future vaccine will not be 100% effective and will provide protection for no more than a few months. We present an individual-based spatially explicit model of wild boar demography and ASF epidemiology, allowing to simulate a vaccination campaign. We tested how many animals should be vaccinated in relation to vaccine efficacy and to the duration of vaccine protection. We estimated how these parameters will affect ASF eradication probabilities. We also assessed how partial vaccination will interact with a series of ecological, epidemiological, and management-related factors linked to ASF persistence. In the case of a highly effective vaccine with short duration, eradication chances were generally low, and the virus disappeared only when simulating a high effort (2.5 vaccinated wild boars/km^2^). A vaccine with low efficacy and long duration was even less effective in eradicating the ASFV, as none of the simulated scenarios provided acceptable eradication rates. Our results indicate that, under realistic conditions, vaccination against the ASF genotype II virus cannot be seen as an effective stand-alone tool for eradication. Its use should be integrated into a more comprehensive strategy, making use of all the available management tools, such as density control through hunting and carcass removal. If the vaccine will exhibit 12 months or longer duration of its protection, splitting the vaccination effort into two or three bait distribution campaigns during the year will be a feasible option. If the vaccine will exhibit a duration of its immunization significantly shorter than 1 year, a single distribution at the end of winter will maximize the probability of eradication.

## 1. Introduction

Since its first introduction into Eurasia in 2007 [1, 2], African Swine Fever (ASF) genotype II has shown a clear tendency to persist in wild boar (*Sus scrofa*) populations [3]. This happened despite the efforts by most of the affected countries to reduce virus circulation through fencing and zone-based restrictions [4] and to make virus transmission less likely by reducing wild boar densities [5]. The few successful eradication examples occurred in Belgium and Czech Republic [6, 7], where the ASF virus was detected in the very early stages of its invasion. In those cases, quick and effective confinement measures were implemented before the affected area became too large to be managed. In all the other instances, ASF persisted and became endemic [8], normally at low or very low virus prevalence levels [9]. This epidemiological pattern makes ASF surveillance activities difficult and often unreliable [10].

Even though ASF poses no threat to human health [11], it causes severe direct and indirect economic losses and has the potential to disrupt the economic system of pork meat production worldwide [12]. At the global level, pork production decreased since the ASF arrival to Europe and Asia, with a consequent sharp increase in meat prices [13].Therefore, although challenging from a theoretical point of view, ASF eradication and control are a priority from both an economic and a food security point of view [14].

As for classical swine fever [15, 16], the availability of a vaccine and its use for mass vaccination could be the key for achieving the eradication of the ASF genotype II virus in infected Eurasian wild boar populations. This would eliminate the main risk factor (infected wild boar populations in sympatry with the domestic pig) for virus transmission to the pig. Given the urgent need to tackle the ASF impact on pig industry, in recent years, several international research groups have started experimental projects to develop an effective and safe vaccine for the ASF genotype II. Up to 2020, a minimum of six different experimental live attenuated vaccines had been tested by different research groups [17], all of them suffering from low levels of protection against the disease. More recent works [18, 19] reported promising figures, with 80%–92% protection against challenge with a virulent ASF virus isolate in domestic pigs, associated with some safety concerns due to the risk of disease development in the case of overdosing [20]. The duration of vaccine protection also varied among the different experimental trials, ranging from a few weeks to a maximum of 5 months [19, 21]. At present, no effective and safe ASF vaccine is available, yet, but some promising experimental vaccine candidates have been proposed by Tran et al. [22]. Still, based on the results of the clinical trials on vaccine candidates, it is realistic that an ASF vaccine will not be 100% effective and will provide protection for no more than a few months.

Given these premises, and due to the theoretical complexity of designing a vaccination strategy on wild populations, there is a need to understand how the characteristics of a possible future ASF vaccine will interact with the other factors affecting disease persistence in wild boar. These factors are population density, temperature-related virus persistence in the environment, and the different management actions used in conjunction with vaccination. It is crucial to evaluate if ASF eradication through vaccination will still be a realistic option in a scenario of partial and temporary vaccination, and how a vaccination program should be designed to maximize eradication chances.

Here, we present an individual-based spatially explicit model of wild boar demography and ASF epidemiology, extended with the option of simulating different types of vaccination strategies, based on vaccines with different characteristics. The model is based on the following assumptions: (a) the vaccine is orally administered, (b) the vaccine confers immunity against the ASF genotype II, (c) the resulting immunity—albeit of variable duration—does not allow infection with the homologous virus and is therefore fully protective, and (d) the vaccine is DIVA (differentiating infected from vaccinated animals), i.e., there is a serological test that can discriminate the presence of vaccine antibodies from those induced by the wild virus.

We first tested how many animals should be vaccinated in relation to vaccine efficacy and to the duration of vaccine protection. We estimated how these parameters would affect ASF eradication probabilities in wild boar. Then, we assessed how partial vaccination is likely to interact with a series of other ecological, epidemiological, and management-related factors linked to ASF persistence. Finally, we present a few realistic options for maximizing ASF eradication probabilities under a scenario of partial and temporary vaccination.

## 2. Methods

**Figure 1:**
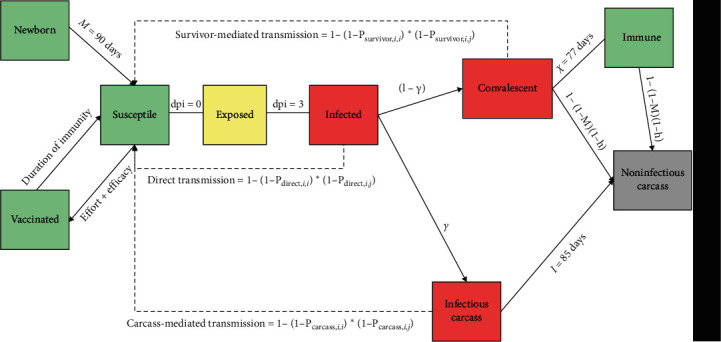
Epidemiological compartments used to build the spatially explicit, stochastic, individual-based model to test different ASF vaccination campaigns on a wild boar population. Red compartments indicate the infectious individuals, green compartments refer to the immune individuals, and grey and yellow indicate the noninfectious dead boars and exposed boars, respectively. The following notations are used for model parameters: *γ* = disease lethality; *χ* = convalescents infectious period; *I* = carcass infectious period; *M* = natural mortality rate; and *h* = hunting rate.

To explore the different scenarios related to an ASF vaccination campaign, we expanded a previously developed and published individual-based model of wild boar demography and ASF epidemiology [23], whose structure and parameterization are shown in Figure 1. First, we added an extra compartment to the model, in which we included vaccinated wild boar. We set the infection probability for vaccinated individuals to zero (Figure 1). As a future ASF vaccine was not expected to be 100% effective nor to provide lifelong protection, we added two more parameters to the model: (1) a probability to develop antibodies and gain protection once a vaccine was ingested (vaccine efficacy) and (2) a duration of protection, after which an individual would be transferred back to the susceptible compartment. Then, to account for the effect of maternal protection, we added a further compartment, in which we placed all piglets born from a vaccinated mother. For this group of individuals, we also set the infection probability to zero and a duration of the maternal protection of 90 days.

We ran the model in Netlogo 6.2.2 on a theoretical initial population of 2,700 wild boar, inhabiting a 900 km^2^ area. This corresponded to a prereproductive density of 3 individuals/km^2^. The prereproductive phase was followed by a postreproductive phase, in which the population density increased, before decreasing again through the action of natural, disease induced, and hunting mortality. We simulated that the vaccination strategy would take place once ASF had reached its endemic condition, with a 1% endemic virus prevalence and a 2% seroprevalence. We simulated a 3-year vaccination effort after a first burn-in year, which only allowed parameter stabilization after the model was initialized. The other parameters were derived from the model version published in [23], which refers to the field data collected in the Baltic countries.

As a first baseline scenario, we ran the model under the theoretical unrealistic hypothesis that a 100% effective vaccine with lifelong protection would be employed. This scenario only served as a benchmark, to be compared with the other, more realistic ones. After generating such baseline scenario, we compared it with three more realistic situations. In the first case, we simulated a vaccine with high efficacy (75%) but short duration (90 days); in the second scenario, we explored the performance of a vaccine with low efficacy (25%) but with a long duration of immunity (365 days); and finally, we tested a compromise vaccine, with 50% efficacy and 180 days of immunity. In all cases, we ran the model under two subscenarios of different vaccination effort. As the vaccine is administered orally, and the same individual is likely to ingest more than one vaccine dose at a time, it was not easy to simulate how many vaccine doses were necessary to vaccinate a single wild boar. The population dynamics of wild boar are extremely variable and consequently the number of individuals in the population fluctuates considerably during the year. Therefore, we did not define a percentage of wild boars to be vaccinated, but we used the measure “no. of vaccinated wild boars/km^2^/year” as an index of vaccination effort. In the first scenario, we simulated an effort of 1.5 vaccinated wild boar/km^2^/year, whereas in the second scenario, the number of vaccinated animals was 2.5/km^2^/year. Then, we calculated the absolute number of vaccinations to be performed each year, as the product between vaccination effort and the size of the simulated study area (900 km^2^), which resulted in 1,350 and 2,250, respectively. We kept the yearly number of vaccinated wild boar constant, irrespective of the small fluctuations in wild boar density occurring during the 3-year period of the vaccination campaign. Finally, for each scenario, we tested two possible temporal patterns of vaccination. In the first case, we divided vaccination effort into three sessions, distributed during the year: one in June, one in September, and one in December. In this case, one-third of the total number of vaccinations, calculated as described above, was administered during each session. In the second case, vaccination effort was concentrated into a single session in January and February, during which all the vaccinations occurred. We ran each combination of parameters over 100 iterations and recorded if eradication occurred, the day of eradication, the daily disease prevalence, the daily proportion of vaccinated individuals who developed immunity, and the daily proportion of ASF seropositive individuals (ASF survivors). We also calculated an average proportion of vaccinated wild boar each year. This proportion was calculated starting from the first day of the vaccination campaign until the end of the year.

After exploring these discrete scenarios, we performed a more comprehensive analysis of the effect of several ecological, management-related, and epidemiological parameters on the likelihood to eradicate ASF through a wild boar vaccination campaign. In each iteration, we randomly extracted parameter values from a uniform distribution in the following ranges:

1. number of vaccinated wild boar: 0%–100% of the postreproductive population
2. vaccine efficacy: 25%–100%
3. duration of vaccine protection: 3 months to 3 years
4. wild boar density (prereproductive): 2–6 individuals/km^2^
5. carcasses removal rate (based on a 60-day effort at the end of winter): 0%–60%
6. annual hunting rate: 0%–50%

We ran the model over 10,000 iterations. For each iteration, we recorded if ASF was eradicated during the 3-year vaccination period and, in that case, the day of eradication. Then, we built and analysed a multinomial logistic regression model using the package *mlogit* in R, in which the response for each iteration was a four -level factor variable corresponding to four possible outcomes (no eradication and eradication within 1, 2, and 3 years). We used all the variables illustrated above as predictors in the regression model. The variables were standardized to make the resulting regression coefficients comparable. The results of the regression model allowed to evaluate the relative importance of the different parameters on the likelihood to eradicate ASF using vaccines.

Finally, based on the results of the comprehensive scenarios exploration, we identified how a successful vaccination strategy should be designed in term of the number of yearly vaccination sessions, total vaccination effort, additional effort in carcass removal, etc., depending on the expected vaccine efficacy and duration of the immunity.

## 3. Results

In the theoretical scenario with full protection and lifelong duration, the ASF vaccine was highly effective under all the simulated scenarios (scenarios 1–4 in Table 1). ASF eradication probability was higher than 90% within 2 years and 100% in 3 years, under all vaccination conditions (Table 1). The average eradication day was in the range of 387–435 (calculated only on the iterations leading to eradication).

**Figure 2:**
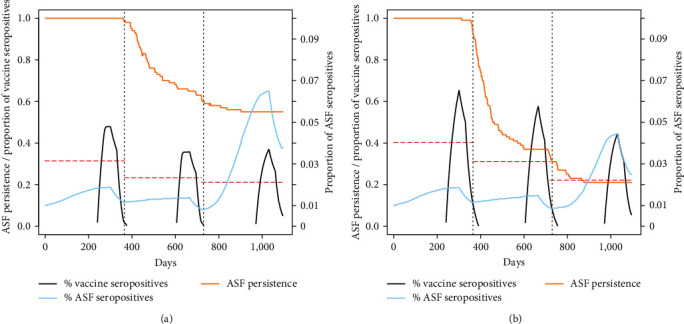
ASF persistence probability (yellow line) during a 3-year vaccination campaign, under the hypothesis of a 75% effective vaccine with a 90-day protection. The black line indicates the proportion of individuals with ASF antibodies, whereas the dashed red lines indicate the annual average of the same parameter. The blue lines indicate the proportion of ASF seropositive individuals (ASF survivors) in the population. (a) Vaccines/km^2^/year = 1.5, efficacy = 75%, and duration = 90 days. (b) Vaccines/km^2^/year = 2.5, efficacy = 75%, and duration = 90 days.

When simulating more realistic scenarios, though, the performances of the vaccination campaigns were less satisfactory. In the case of a highly effective vaccine with short duration (scenarios 5–8 in Table 1), eradication chances were high (>90%) only when simulating a high effort (2.5 vaccinated wild boars/km^2^) concentrated in a single session in late winter. In all the other simulated scenarios, ASFV persistence probability remained high for the whole 3-year period. The low effectiveness of this type of vaccine was witnessed also by the relatively low proportion of vaccinated individuals who developed immunity in the population, which remained always below 40% (Figure 2) and by the increasing proportion of ASF seropositive individuals due to a high virus circulation in the population (Figure 2). The average eradication day under this group of scenarios was in the range of 539–906 (Table 2).

**Figure 3:**
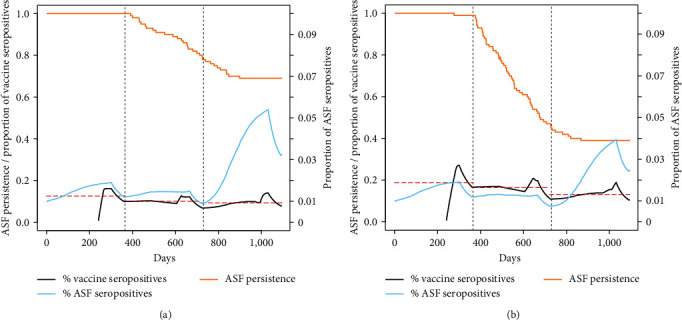
ASF persistence probability (yellow line) during a 3-year vaccination campaign, under the hypothesis of a 25% effective vaccine with a 365-day protection. The black line indicates the proportion of individuals with ASF antibodies, whereas the dashed red lines indicate the annual average of the same parameter. The blue lines indicate the proportion of ASF seropositive individuals (ASF survivors) in the population. (a) Vaccines/km^2^/year = 1.5, efficacy = 25%, and duration = 365 days. (b) Vaccines/km^2^/year = 2.5, efficacy = 25%, and duration = 365 days.

A vaccine with low efficacy and long duration (scenarios 9–12 in Table 1) was even less effective in eradicating ASF. None of the simulated scenarios provided acceptable eradication rates, with ASF persistence being higher than 40% after 3 years of vaccination. The proportion of vaccinated individuals who developed immunity was <20%, and there was a sharp increase in the proportion of ASF seropositive wild boars (Figure 3). The average eradication day under this group of scenarios was in the range of 775–975 (Table 2). Finally, a compromise vaccine (scenarios 13–16 in Table 1) performed slightly better, especially when used in combination with a high vaccination effort concentrated in a single session in winter. Under this scenario, the vaccine provided about 90% eradication chances after 3 years, with the average eradication day being in the range of 561–887.

The results of the multinomial regression model indicated that vaccination effort, expressed as the number of vaccinations per square kilometer, was the most important variable affecting the probability to eradicate ASF (*β* = 3.99; SE = 0.06 for year 1; Table 2). The second most important parameter correlating with eradication was vaccine efficacy (*β* = 1.66; SE = 0.04 for year 1), followed by the number of vaccination sessions during the year (*β* = −1.24; SE = 0.04 for year 1). This parameter was negatively correlated with eradication rates, suggesting that a more concentrated vaccination effort in time provided a higher chance to achieve ASF eradication. The duration of immunity was positively correlated with eradication, but its associated regression coefficient was lower than the ones associated with the other parameters described above (*β* = 0.55; SE = 0.03 for year 1). The model also indicated that the likelihood of ASF eradication was lower when performed on a wild boar population living at higher density, as witnessed by the negative and significant regression coefficients estimated for that variable (Table 2). Finally, both the removal of infected carcass and the use of hunting to control population density were significantly related to an increase in the chances of ASF eradication, but the magnitude of their effect was smaller than that associated with the most important parameters, such as effort and efficacy (Table 2).

**Figure 4:**
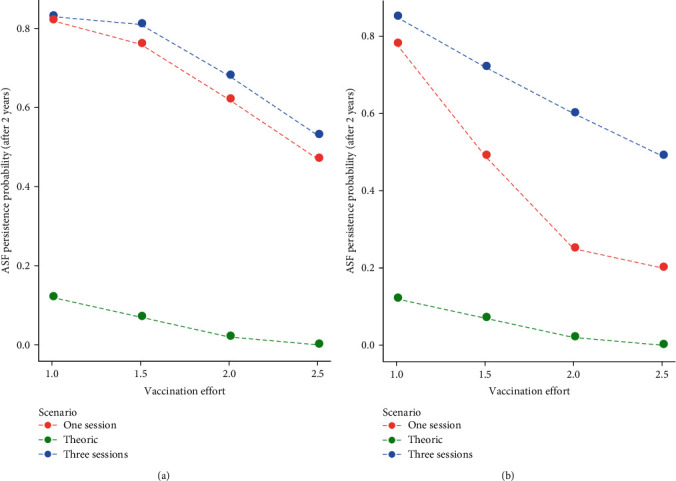
Relationship between vaccination effort and ASF persistence probability (after 2 years of vaccination) when using: (a) a vaccine with low efficacy (25%) and long duration (365 days) and (b) a vaccine with high efficacy (75%) and short duration (90 days). In both cases, the performances of each vaccine are compared with those of a theoretical 100% effective lifelong vaccine (green line) under two scenarios: (1) a campaign with three 30-day yearly sessions (blue line) and (2) a campaign with a single 60-day yearly session (red line).

When plotting the relationship between vaccination effort and ASF eradication rates for all the simulated scenarios (Figure 4), we were able to put in evidence a clear difference between the different types of simulated vaccines. When simulating with a long duration of the immunity (365 days), the number of vaccination sessions did not affect eradication rates (Figure 4(a)). In contrast, when simulating a highly effective (75%) with short duration of the immunity (90 days), an increasing vaccination effort was more effective when applied in a single session in winter (red line in Figure 4(b)) rather than in three sessions along the year (blue line n Figure 4(b)). This suggests that the optimal temporal distribution of vaccines was dependent on vaccine characteristics.

**Figure 5:**
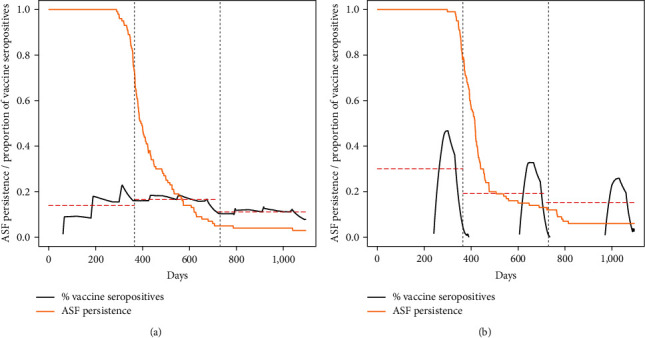
ASF persistence probability (yellow line) during a 3-year vaccination campaign, under two realistic scenarios providing a high probability to eradicate ASF within 3 years. (a) A 50% effective vaccine with a 365-day protection, distributed in three 30-day sessions each year with an effort of 1.5 vaccinations/km^2^, combining vaccination with a 50% removal of infected carcasses in late winter and a 30% annual hinting rate and (b) a 75% effective vaccine with a 90-day protection, distributed in a single 60-day session each year in winter with an effort of 1.5 vaccinations/km^2^, combining vaccination with a 50% removal of infected carcasses in late winter and a 30% annual hinting rate. The black line indicates the proportion of individuals with ASF antibodies, whereas the dashed red lines indicate the annual average of the same parameter. (a) Vaccines/km^2^/year = 1.5, efficacy = 50%, and duration = 365 days. (b) Vaccines/km^2^/year = 1.5, efficacy = 75%, and duration = 90 days.

Based on the full parameter exploration and results of the discrete scenarios, we defined two possible vaccination campaigns with a high probability to achieve ASF eradication within 3 years, based on the different characteristics of a future ASF vaccine. If a vaccine with a 50% efficacy and a 1-year duration of its protection was developed, such vaccine should be distributed with an effort of 1.5 vaccinated wild boar/km^2^ in a population with a density of 3 wild boar/km^2^, i.e., one vaccination for each two individuals in the population (prereproductive density). This vaccine should be distributed in three sessions during the year, combined with a 50% carcass removal in late winter, and a 30% annual hunting rate. As shown in Figure 5(a), this vaccination campaign would provide a very high ASF eradication probability after 2 years.

If a vaccine with a 75% efficacy and a 90-day duration of its protection should be developed, such vaccine should be distributed with an effort of 1.5 vaccinations/km^2^ in a population with a density of 3 wild boar/km^2^. In this case, the vaccination campaign should be organized into a single session in January to February, combined with a 50% carcass removal in late winter, and a 30% annual hunting rate. As shown in Figure 5(b), this vaccination campaign would also provide a very high ASF eradication probability after 2 years. Based on the preliminary tests on candidate vaccines published so far, this second scenario of high efficacy and short duration seems to be the most likely for the immediate future.

## 4. Discussion

Our analysis confirms that the characteristics of a future vaccine for the ASF genotype II virus will have important consequences both on the likelihood to eradicate the disease in wild boar populations and on the way a vaccination campaign should be planned and realized. Under the unlikely scenario of a highly effective vaccine with a multiyear duration of its protection, our model indicated that vaccination could be highly effective, to the point of being used as a stand-alone eradication tool. In such a case, at least 25% of the population should be vaccinated in the first year and the same proportion in the following year, allowing to reach a 50% coverage of the population in 2 years (Table 1). This scenario has several similarities with the classical swine fever (CSF) eradication experience in the late 90′s of last century and early years of this century [15]. In that case, the live-attenuated vaccines employed in the vaccination campaign exhibited a high effectiveness and a long duration of protection up to 10 months after bait ingestion [24]. Under those conditions, vaccination often resulted in disease eradication, especially when the infected area was relatively small and the proportion of vaccinated individuals in the population was around or higher than 50% [15].

The early experimental studies on the ASF genotype II vaccine candidates, though, draw quite a different picture, as both the effectiveness and the duration of protection are expected to be lower than in the CSF case [19, 21]. Under these more realistic scenarios, simulated vaccination campaigns often ended up in a failure, especially when used alone (Table 1). The few instances in which ASF was eradicated were the result of a massive field effort, in which at least 40% of all the individuals in the population were vaccinated already during the first year of the campaign. This indicates that, under realistic conditions, vaccination against the ASF genotype II virus cannot be seen as an effective stand-alone tool for eradication, but its use should be integrated into a more comprehensive strategy, making use of all the available management tools, such as density control through hunting and carcass removal.

In particular, a careful use of wild boar hunting should be integrated in the eradication strategy, because of the need to keep wild boar densities relatively low during the eradication effort and to prevent an increase in wild boar encounter and infection rates, which could jeopardize the effect of vaccination. It will be crucial, though, to avoid any spatial correlation between vaccination probability and hunting probability, which would result in vaccinated individuals having a higher probability to be shot than nonvaccinated individuals. In CSF vaccination campaigns, baits were usually provided by hunters at feeding grounds [15], but hunting was often stopped 1 week before and during bait distributions, to avoid animal disturbance and to limit the risk of false polymerase chain reaction (PCR)-positive results [25].

Our analyses also showed that the temporal structure of the vaccination campaign should be adapted to vaccine characteristics (Figure 4(a)). If the vaccine will exhibit long duration of its protection (about 12 months or longer), splitting vaccination effort into two or three bait distribution campaigns during the year [15] will be an option that will relax the effort needed at each distribution, while providing high eradication probabilities. This would be in line with what done in most of the CSF vaccination campaigns [15], in which the long duration of the immunization allowed to provide baits three times a year. In contrast, if the vaccine will exhibit a low duration of its immunization effect (significantly shorter than 1 year), our simulations showed that a single distribution at the end of winter will maximize the probability of eradication (Figure 4(b)). Previous modelling work [26] has shown that the period immediately before recruitment at the end of winter is the one during which it is more likely to eradicate ASF through hunting and carcass removal. Therefore, in case a single vaccination campaign should be performed during the year, providing baits in the same 2-month window at the end of winter would allow to combine the effects of all the available eradication strategies and to maximize the probabilities of achieving an early ASF eradication from the wild boar population. The application of an integrated eradication strategy, though, will imply a massive effort to allow an almost simultaneous deployment of baits in a network of feeding stations, the intensive search and removal of infected carcasses, and the monitoring of the wild boar population after the completion of the vaccination campaign. This will be possible only through an effective involvement of the stakeholders including hunters, wildlife agencies, local and central veterinary services, etc.

Finally, it should be noted that our simulation model was based only on a quantification of successful vaccination events (bait ingestion by a wild boar) and on a uniform spatial distribution of vaccination probability. This represents a simplification of reality. Previous vaccination campaigns have shown that landscape structure and animal movement patterns influence the probability by each wild boar to get in contact with vaccine baits [27] and that vaccination fields should be distributed at distances and with a spatial design which allow most of the individuals in the population to get access to baiting fields. Moreover, a large amount of vaccine baits should be provided per area unit, to consider the possibility of bait loss and multiple bait ingestions by the same individual. In CSF vaccination, the spatial design corresponded to the delivery of about 40 baits per vaccination ground and a density of one to two vaccination grounds per square kilometer of forest [15]. In the case of ASF, specific studies should be conducted, considering landscape structure, wild boar home range size, and space use, to identify a specific vaccination design suited for the type of vaccine available and the characteristics of the affected wild boar population.

## Figures and Tables

**Table 1:** Summary statistics resulting from a set of simulated ASF vaccination scenarios.

No.	Vaccine type	Vaccination effort ^*∗*^	Efficacy	Duration (days)	No. of sessions	Proportion of vaccine seropositive individuals			ASF persistence probability			Average eradication day
						Year 1	Year 2	Year 3	Year 1	Year 2	Year 3	
1	Theoretical	1.5	1	Lifetime	3	0.29	0.47	—	0.66	**0.07**	**0**	387
2		2.5	1	Lifetime	3	0.45	0.54	—	*0.20*	**0**	**0**	331
3		1.5	1	Lifetime	1	0.47	0.43	0.39	0.92	**0.01**	**0**	435
4		2.5	1	Lifetime	1	0.52	0.48	—	0.86	**0**	**0**	407

5	Highly effective short duration	1.5	0.75	90	3	0.12	0.09	0.09	0.96	0.72	0.68	906
6		2.5	0.75	90	3	0.19	0.14	0.14	0.89	0.49	0.47	756
7		1.5	0.75	90	1	0.31	0.21	0.23	0.97	0.49	0.44	784
8		2.5	0.75	90	1	0.40	0.30	0.23	0.92	*0.20*	**0.08**	539

9	Lowly effective long duration	1.5	0.25	365	3	0.07	0.09	0.09	0.98	0.81	0.80	975
10		2.5	0.25	365	3	0.12	0.15	0.14	0.93	0.53	0.48	809
11		1.5	0.25	365	1	0.12	0.10	0.09	1.00	0.76	0.70	934
12		2.5	0.25	365	1	0.19	0.16	0.13	0.96	0.47	0.42	775

13	Effectiveness–duration compromise	1.5	0.5	180	3	0.12	0.10	0.10	0.94	0.68	0.68	887
14		2.5	0.5	180	3	0.20	0.16	0.14	0.86	0.32	0.28	647
15		1.5	0.5	180	1	0.24	0.17	0.15	1.00	0.54	0.48	805
16		2.5	0.5	180	1	0.35	0.25	0.20	0.99	*0.18*	**0.10**	561

^*∗*^Vaccination effort is indicated as the number of vaccinations in each square kilometer each year. Vaccines with different efficacy and duration of protection were tested and compared under two possible vaccination designs (a single annual vaccination campaign vs. three campaigns). The figures highlighted in bold indicate the scenarios corresponding to an ASF persistence probability <0.1, whereas the ones highlighted in italic to a persistence probability >0.1 and <0.2.

**Table 2:** Estimates of the regression coefficients derived from a multinomial regression model, in which the year of ASF eradication was used as response variable and a set of ecological, management-related, and epidemiological parameters as predictors.

Parameter	Level	Estimate	SE	*P*-value
Vaccination effort	Year 1	3.99	0.06	<0.01
	Year 2	3.18	0.06	<0.01
	Year 3	1.92	0.08	<0.01

Vaccine efficacy	Year 1	1.66	0.04	<0.01
	Year 2	0.80	0.03	<0.01
	Year 3	0.23	0.05	<0.01

No. of sessions	Year 1	−1.24	0.04	<0.01
	Year 2	−0.44	0.03	<0.01
	Year 3	−0.42	0.04	<0.01

Duration of immunity	Year 1	0.55	0.03	<0.01
	Year 2	0.54	0.03	<0.01
	Year 3	0.57	0.04	<0.01

Hunting rate	Year 1	0.44	0.03	<0.01
	Year 2	0.34	0.03	<0.01
	Year 3	0.23	0.04	<0.01

Carcass removal rate	Year 1	0.41	0.03	<0.01
	Year 2	0.21	0.03	<0.01
	Year 3	0.09	0.04	0.02

Wild boar density	Year 1	−0.42	0.03	0.03
	Year 2	−0.38	0.02	0.02
	Year 3	−0.27	0.03	0.04

All variables were standardized, so that the regression coefficients are comparable and shown in order of importance.

## Data Availability

Data sharing is not applicable to this article as no new data were collected for this study.
